# Editorial: Effects of economic shocks on human behavior, mental life and the environment: Implications for the post-COVID-19 crisis era

**DOI:** 10.3389/fpsyg.2022.922875

**Published:** 2022-07-19

**Authors:** Giray Gozgor, Chi Keung Marco Lau, Jie Ma

**Affiliations:** ^1^Faculty of Political Sciences, Istanbul Medeniyet University, Istanbul, Turkey; ^2^School of Business, Hang Seng University of Hong Kong, Hong Kong, Hong Kong SAR, China; ^3^Newcastle Business School, Northumbria University, Newcastle upon Tyne, United Kingdom

**Keywords:** the COVID-19 pandemic, economic shocks, human behavior, business transformation, financial markets

The COVID-19 pandemic has imparted fundamental economic shocks to society. Indeed, the COVID-19 pandemic is a threat to individual health and survival. Its onset changes human behavior in both the short- and long run. This issue may lead to changes in human behavior and affect mental life. The lifestyle change during the COVID-19 pandemic has also increased depression and suicide. The COVID-19 pandemic requires an integrated effort from governments, institutions, organizations, and communities to combat this health crisis.

The “CoVesity” issue conflated the rise in obesity with the various measures (e.g., lockdowns, curfews, social distancing), which significantly reduced human activity, mobility, and hence exercise (Zakka et al., 2021; Ertz and Le Bouhart, 2022). In addition, some studies attempted to further detail how it might be possible to take advantage of the pandemic to reach a more sustainable society. In the following, a consortium of researchers used several theoretical frameworks to suggest a triple-layered (systemic) shift toward more sustainable societies: (1) proactive politics funded over time (decision-makers); (2) agile and socially responsible organizations; (3) agentic and empowered citizens (consumers) (Trespeuch et al., 2021).

The nature of businesses everywhere is also transforming in the aftermath of this global pandemic; for example, online working from home is increasingly adopted by companies wishing to reduce fixed costs and minimize organizational risk. There will also be significant changes in networking and social interactions. These issues affect the workers' emotions, mental fatigue, and stress. In addition, on a societal level, people's attitudes toward one another may be transformed, as exemplified by voluntary groups' growth to support vulnerable community members. For instance, good neighborliness and altruism exert pressure for change in government and businesses, especially in free-market economies, where economic considerations are now beginning to dominate the debate. Given these backdrops, this research topic contains 18 papers on the effects of economic shocks on human behavior, businesses and financial markets.

Regarding papers focusing on individuals' behavior, Rojas-Méndez uses the data for more than 24,000 individuals living in 30 different countries and finds that socio-economic variables and cultural dimensions are essential in explaining people's concerns about the adverse effects of the COVID-19 pandemic. Song et al. show that China's New Rural Cooperative Medical System increases the Chinese people's happiness by promoting poverty alleviation and rural revitalisation. The system will help combat the adverse outcomes of the COVID-19 pandemic. Lu and Lin discuss the economic and social consequences of the COVID-19 pandemic and offer a conceptual analysis of how the COVID-19 pandemic affects individual mental health and coping behaviors from the perspectives of personal social contexts. In addition, Ai et al. find that physical exercise decreases people's anxiety, sadness, and depression during the COVID-19 pandemic. Gong et al. review the effects of the declining global gross domestic product due to the outbreak of the COVID-19 pandemic on mental problems. Xie et al. obtain valuable evidence for policymakers fighting against the COVID-19 pandemic and find that the self-construction methods explain wellbeing with the different consumption choices.

Several papers have focused on the business aspect of the COVID-19 pandemic. Liu et al. discuss that cross-culture conflict management is an essential issue for Chinese firms during the COVID-19 pandemic. Wu and Zhu suggest that Chinese firms' Corporate Social Responsibility engagement policies fight against the negative consequences of the COVID-19 pandemic. Ma et al. review the effects of the COVID-19 pandemic on global inequality and find the persistency in inequality among countries' service sectors. Ma et al. also show that the service sector is the most significant contributor to global inequality, which may be substantial evidence for the post-COVID-19 era. According to Ma et al., there has been a considerable decline in global inequality from 2000 to 2017, and East Asian economies have contributed almost 40% of this decline in global inequality. Hu et al. indicate that the lockdown during the COVID-19 pandemic led to a short-run reduction in carbon dioxide emissions in 30 Chinese regions in 2020. Wang implements 210 valid questionnaires for employees in different Chinese enterprises and shows that the dynamic work environment positively affects innovative performance during the COVID-19 pandemic. Chen et al. discuss the effects of the big data capability on financial performance in Chinese firms in the context of the digital economy explosion during the COVID-19 pandemic.

Several papers have also analyzed the effects of the COVID-19 pandemic on financial markets. For instance, Jiang et al. observe that investors' sentiment in China (measured by the Baidu index) is crucial for predicting stock trends in China. The evidence may be helpful for the COVID-19 pandemic. Song et al. also show that investor sentiment is an essential determinant of the stock market returns of the newly listed Chinese companies from October 2019 to June 2020. Sun et al. obtain the negative impact of the COVID-induced public anxiety, measured by Google Trends and Wikipedia data, on 14 European stock markets from January 2, 2020, to September 17, 2020. Lu et al. find that the COVID-19 pandemic caused a significant increase in the risk perceptions for the stock market performance of the United States airline companies.

In short, this research topic covers 18 papers on the consequences of the COVID-19 pandemic with its effects on individuals, businesses, and financial markets (especially the stock market returns).

## Author contributions

GG writing and reviewing the manuscript. CL and JM writing the manuscript. All authors contributed to the article and approved the submitted version.

## Conflict of interest

The authors declare that the research was conducted in the absence of any commercial or financial relationships that could be construed as a potential conflict of interest.

## Publisher's note

All claims expressed in this article are solely those of the authors and do not necessarily represent those of their affiliated organizations, or those of the publisher, the editors and the reviewers. Any product that may be evaluated in this article, or claim that may be made by its manufacturer, is not guaranteed or endorsed by the publisher.
